# POTN: A Human Leukocyte Antigen-A2 Immunogenic Peptides Screening Model and Its Applications in Tumor Antigens Prediction

**DOI:** 10.3389/fimmu.2020.02193

**Published:** 2020-10-07

**Authors:** Qingqing Meng, Yahong Wu, Xinghua Sui, Jingjie Meng, Tingting Wang, Yan Lin, Zhiwei Wang, Xiuman Zhou, Yuanming Qi, Jiangfeng Du, Yanfeng Gao

**Affiliations:** ^1^ School of Life Sciences, Zhengzhou University, Zhengzhou, China; ^2^ School of Pharmaceutical Sciences (Shenzhen), Sun Yat-sen University, Shenzhen, China

**Keywords:** neoantigen prediction, peptides, immunogenicity, prediction model, cancer immunotherapy

## Abstract

Whole genome/exome sequencing data for tumors are now abundant, and many tumor antigens, especially mutant antigens (neoantigens), have been identified for cancer immunotherapy. However, only a small fraction of the peptides from these antigens induce cytotoxic T cell responses. Therefore, efficient methods to identify these antigenic peptides are crucial. The current models of major histocompatibility complex (MHC) binding and antigenic prediction are still inaccurate. In this study, 360 9-mer peptides with verified immunological activity were selected to construct a prediction of tumor neoantigen (POTN) model, an immunogenic prediction model specifically for the human leukocyte antigen-A2 allele. Based on the physicochemical properties of amino acids, such as the residue propensity, hydrophobicity, and organic solvent/water, we found that the predictive capability of POTN is superior to that of the prediction programs SYPEITHI, IEDB, and NetMHCpan 4.0. We used POTN to screen peptides for the cancer-testis antigen located on the X chromosome, and we identified several peptides that may trigger immunogenicity. We synthesized and measured the binding affinity and immunogenicity of these peptides and found that the accuracy of POTN is higher than that of NetMHCpan 4.0. Identifying the properties related to the T cell response or immunogenicity paves the way to understanding the MHC/peptide/T cell receptor complex. In conclusion, POTN is an efficient prediction model for screening high-affinity immunogenic peptides from tumor antigens, and thus provides useful information for developing cancer immunotherapy.

## Introduction

Cancer immunotherapy has achieved great success in several cancer types (1–3), although durable clinical responses only occur in some patients. Evidence from patients who responded to immunotherapy suggests that tumor regression is achieved by activating tumor-antigen-specific CD8^+^ cytotoxic T lymphocytes (CTLs) (4–7). Tumor antigens are generated by tumor-specific proteins (8) and presented by the formation of peptide/major histocompatibility complex (MHC)-I complexes on cell surfaces *via* antigen presentation (9).

Generally, tumor antigens can be classified as tumor-specific antigens, including neoantigens, and as tumor-associated antigens. Neoantigens are exclusively presented on tumor cell surfaces, whereas tumor-associated antigens are highly expressed on tumor cells but are also expressed on normal cells at a low level. Using patients’ specific neoantigens as tumor vaccines is a safe, feasible approach to eliciting a clinical T cell response (4). However, studies on a large-scale peptide collection found that only about 1% of the peptides can bind MHC-I molecules (10), and less than 0.3% of the peptides should be validated experimentally for immunogenicity (11). We still lack knowledge about the key features of immunogenic peptides and efficient methods to screen tumor antigen peptides from a large number of tumor mutations in personalized immunotherapy.

Tumor antigens can be identified by several approaches. Screening tumoral cDNA libraries with phage display is a powerful but labor-intensive approach to identifying tumor-associated antigens (12–14). Exome sequencing of tumor biopsy and paired normal tissues have been widely applied to screening the mutated fragments (15, 16). The fragments can be synthesized experimentally and tested further for their antigen presentation by measuring the MHC binding affinity, and for their immunogenicity *via* ELISpot, intracellular cytokine staining (ICS), and human leukocyte antigen (HLA) tetramers (15). Another approach to identifying tumor antigens is based on mass spectrometry, which identifies the sequence of peptides presented on the tumor cell surface by MHC molecules (17–19).

Reliable predictions of antigenic peptides from high-throughput sequencing data can lighten the experimental burden for identifying epitopes. *In silico* prediction programs have been developed for this purpose. For example, NetChop and ProteaSMM analyze the proteasomal cleavage pattern and the antigen processing mechanism (20–22), while NetMHCpan 4.0 and other programs predict epitopes by calculating the binding affinity of peptide/MHC allele complexes (23, 24). Other programs use a combined algorithm that integrates proteasomal cleavage prediction, the transporter associated with antigen processing (TAP) transport efficiency, and MHC binding affinity (25). These programs focus on binding capacity prediction, TAP transport prediction, and proteasomal cleavage prediction. We have used these prediction programs to identify epitopes and we found that for HLA-A2 epitopes, fewer than 20% of the predicted epitopes could induce T cell responses (26–28). Thus, the prediction accuracy of the available software packages still needs to be improved.

There are two main reasons for the limited prediction accuracy of current epitope identification programs. First, most of the programs were developed based on a pan-specific method, which does not differentiate between HLA alleles, and they are widely used to make predictions for various HLA alleles. Therefore, when they are used to identify the antigenic peptides for a particular MHC allele, the accuracy is lower because of their inherent features (29). Second, the datasets used to construct the prediction models in many programs are impure. Non-immunogenic peptides in many datasets are randomly selected and are not experimentally validated, resulting in high false-negative rates. To avoid such shortcomings, we gathered experimental data and built a prediction model for only the most common HLA allele (30). About 5200 HLA-A alleles have been identified, among which HLA-A2 shows a high occurrence; the proportion of people with the HLA-A2 allele is 54.0% in ethnic Chinese people and 43.1% of the general population (30–32).

In this study, we selected 9-mer peptides (nonamers) with verified immunological activity and used a support vector machine (SVM) to construct the POTN prediction model for the HLA-A2 allele based on the physicochemical properties of amino acids. We validated the model by using external data. We used the POTN model to predict immunogenic peptides from the cancer-testis antigen located on the X chromosome (CT-X) and measured the binding affinity and immunological activity by ICS of the predicted peptides. We compared the prediction accuracy of POTN with that of other widely used prediction software. Our model may provide a new method to screen high-affinity immunogenic peptides from amino acid sequences or whole-exome sequencing data efficiently.

## Materials and Methods

### Peptide Data Collection

The immunogenic peptides were retrieved from the databases IEDB (33, 34), SYFPEITHI (35), and Peptide Database (36). To ensure that the dataset was not biased, peptides matching our selection criteria were randomly selected from the databases. From the IEDB database, we obtained 41 HLA-A2 cancer-associated immunogenic peptides using our initial screening criteria for the MHC-I linear epitope. From the SYFPEITHI database, 41 T cell epitopes were obtained by searching for HLA-A2 cancer-associated peptides that did not overlap with the peptides obtained from IEDB. The Peptide Database contains human tumor antigen peptides categorized as mutation, tumor-specific, differentiation, and overexpressed. We selected 64 unique peptides by excluding peptides that overlapped with the peptides from the other two databases. The peptides used as a negative dataset were screened from the IEDB database and the literature, and 214 peptides that were experimentally validated as non-immunogenic peptides were obtained (**Table S1**).

The final dataset consisted of a total of 360 HLA-A2 peptides, including 146 immunogenic peptides and 214 non-immunogenic peptides (**Table 1**). For the total dataset, 60% of the immunogenic peptides and 60% of the non-immunogenic peptides were selected as the training set, and the remaining 40% of the peptides were used as the test set (**Table S1**), where approximately 6% of the dataset were eluted peptides.

**Table 1:** Data collection for the model construction and evaluation.

Resources	T cell response		Total (*n *= 360)
	Yes (*n* = 146)	No (*n* = 214)	
IEDB	41	16	57
Peptide database	64	0	64
SYFPEITHI	41	0	41
Literatures	0	198	198

### Selection and Calculation of Potential Immunogenic Properties

To obtain the most useful properties, we searched the literature to find features that may be relevant to immunogenicity. The accessible surface area (ASA) has been used to understand various biological problems, such as protein-protein interactions (37, 38), structural epitopes (39), and active sites (40), and it was used as a feature to build the model. The polarity and charge of amino acids in a peptide are highly correlated with binding affinity (41, 42), and thus these features were used in model construction. In addition, physicochemical properties, including isotropic surface area (ISA), electronic charge index (ECI), hydrophobicity, entropy, molecular weight (Mw), aromatic residues, organic solvent/water, and isoelectric point (PI), have been studied (7, 43–50). The physicochemical properties of 20 amino acids were obtained from the amino acid index database (51).

The properties for binding, protein cleavage, and TAP transport efficiency of each peptide were calculated by online server NetCTL 1.2 with default parameters (52). The T cell recognition score and the stability of the peptide/MHC complexes were considered (48, 53).

Because some residues tend to be in specific positions in the immunogenic peptides (54), we calculated the residue propensity, which is defined as the probability of an amino acid being at an individual position of a peptide, as

$$\text{Residue propensity }\left({\text{R}}_{i}{\text{P}}_{j}\right)=\frac{{\text{P}}_{j}*100-{\text{N}}_{j}*100}{{\text{P}}_{j}*100+{\text{N}}_{j}*100}$$

where P*_j_* is the frequency of residue *i* at position *j* for immunogenic peptides and N*_j_* is the frequency of residue *i* at position *j* for non-immunogenic peptides.

To understand the discriminative power of predictors better, we calculated the statistical significance (*p*-values) of each predictor for immunogenic peptides versus non-immunogenic peptides in the training set using Student’s *t*-test. Only predictors with significant differences (*p* < 0.05) between immunogenic and non-immunogenic peptides were included in the final model (**Table 2**).

**Table 2:** Selected features for model construction. The selected features were highly correlated with immunogenicity (indicated by *p*-value).

Features	References	Position	Description	*p*-value
ASA	(39)	P3	Accessible surface area	0.026
Charged value	(42)	P3	Net charge	0.039
ECI	(44)	P3	Electronic charge index	0.009
Entropy	(46)	P3	Entropy of formation	0.001
Hydrophobicity	(45)	P3	Modified Kyte-Doolittle hydrophobicity scale, more hydrophobic residues are preferable to be at P4, P7, and P8.	2.35E-05
ISA	(44)	P3	Isotropic surface area	1.62E-06
Mw	(47)	P3	Molecular weight	0.042
Organic solvent/water	(50)	P3	Transfer energy, organic solvent/water	4.58E-05
Organic solvent/water	(50)	P4	Transfer energy, organic solvent/water	0.019
PI	(49)	P5	Isoelectric point	0.028
Polarity	(42)	P3	Polarity	0.002
Residue propensity	(54)	P1	Score based on frequency assigned of each amino acid (see **Figure 1**)	0.000
Residue propensity	(54)	P2		0.003
Residue propensity	(54)	P3		2.11E-14
Residue propensity	(54)	P4		4.73E-06
Residue propensity	(54)	P5		4.10E-07
Residue propensity	(54)	P6		8.73E-05
Residue propensity	(54)	P7		2.57E-06
Residue propensity	(54)	P8		1.41E-06
Residue propensity	(54)	P9		0.000
Residue propensity	(54)	sum		2.84E-41
Aff	(7, 52)		binding affinity	7.64E-08
Aff_rescale	(52)		Rescale binding affinity	7.63E-08
Cle	(7)		C terminal cleavage affinity	0.003
Combined score	(7)		Combined prediction score	3.04E-08
Pred	(53)		pMHC stability score	2.42E-08
Thalf	(53)		pMHC stability score	0.001
NB	(53)		pMHC stability score	1.46E-09

### Construction of the Immunogenic Prediction Model

SVM is a supervised learning model based on the principles of structure risk minimization and the kernel method (55), and it has been widely used to predict T cell epitopes (56). Here, SVM with a radial basis (Gaussian) kernel was used to construct the POTN model based on the selected immunogenicity predictors. The regularization parameter (C), which controls the trade-off between the margin and the training error, was tested for model construction and optimization. In optimizing the model construction, several C values (C ∈{0.25,0.50,1,2,4}) were used to construct the model, and the values were validated by the leave-one-out approach in R (version 3.5.2).

### Peptide Prediction and Synthesis

Candidate peptides from CT-X were predicted using the POTN model and 34 peptides with the highest scores were selected, of which 22 peptides with satisfactory solubility were synthesized by the standard solid-phase Fmoc strategy (57) and purified by reverse phase high-performance liquid chromatography (58). All synthesized peptides had a purity of >95%, as measured by electrospray ionization mass spectrometry.

### Binding Affinity Measurement

The T2 binding assay was used to determine the binding affinity of the candidate peptides and HLA-A2 molecule by using a previously described protocol (27). The T2 cell line (HLA-A2) was supplied by Professor Yuzhang Wu (Third Military Medical University, Chongqing, China). In brief, T2 cells (500 μL, 1 × 10^6^ cells/mL) were incubated with the peptide (25 μg, 50 μg/mL; dissolved in DMSO at a concentration of 10 mg/mL) in serum-free IMDM medium, supplemented with human β2-microglobulin (3 µg/mL, Merck, USA) at 37°C for 18 h. The T2 cells were washed twice and incubated with the anti-human HLA-A2-PE-cy7 antibody (BB7.2, eBioscience, USA) at 4°C for 30 min. The mean fluorescence intensity (FI) of each group was analyzed by flow cytometry (FACSCalibur, Becton-Dickinson, USA). Based on the FI, the binding affinity of the candidate peptides toward HLA-A2 molecule was calculated by

$$\text{FI }=\frac{\text{a}-\text{b}}{b}$$

where a is the mean PE-cy7 FI with the peptide and b is the mean PE-cy7 FI without the peptide.

### ICS Assay for Immunogenicity

We determined whether the high-binding affinity peptides elicited a T cell response in peripheral blood samples from five HLA-A2^+^ healthy donors. The blood samples were obtained from Henan Red Cross Blood Center (Zhengzhou, China) with the approval of the Institutional Ethics Review Board. All research was performed under the approval of the Ethics Committee of Zhengzhou University. An ICS assay was used to quantify IFN-γ production of CD3^+^CD8^+^ T cells. Peripheral blood mononuclear cells (PBMCs) were stimulated by each peptide (10 μg/mL) once-weekly for 3 weeks according to our previous work (59). On day 21, the induced T cells from the PBMCs were used as effector cells, and T2 cells were incubated with the synthesized peptides (50 μg/mL) for 4 h as the stimulator cells. The effector cells (1 × 10^6^) and stimulator cells (1 × 10^6^) were co-incubated for 3 h, and brefeldin A (2 μg/mL, Sigma-Aldrich, USA) was added to block the release of produced cytokines for another 5 h at 37°C and 5% CO_2_. The cells were washed and stained with eFlour 710 labeled anti-human CD3 antibody and APC-labeled anti-human CD8 antibody (eBioscience) for 30 min at 4°C before fixation and permeabilization. Permeabilized cells were intracellularly stained with the PE-labeled anti-human IFN-γ antibody (BioLegend, Inc., USA) for 30 min on ice in the dark. Cells were resuspended in buffer for acquisition and analysis using a flow cytometer (FACSCalibur, Becton Dickinson).

## Results

### Identification of Features and Key Residues for Immunogenic Peptides

Feature selection is a crucial step in model construction. To avoid overlaid features and decrease the less-valuable features in the model, we selected properties that have been linked to immunogenicity. We found that 28 features were significantly different between the immunogenic and non-immunogenic groups of peptides (**Table 2**). Aromatic amino acids were not significantly different at either a single position or a sum of points, and TAP also made no significant difference in our dataset.

By statistically analyzing the differences in the residual properties for each position, we found that many physicochemical properties are significantly different at position 3 (P3) between the immunogenic peptides and the non-immunogenic peptides, which has not been reported before (**Table 2**) (60). Thus, we hypothesized that the residues at P3 should be small and flexible, which may contribute to the binding of P4–P7 to the MHC/peptide/T cell receptor complex (61). To test our hypothesis, we screened for pairs of peptides with only one amino acid different at P3, where one peptide was immunogenic and the other was non-immunogenic. We found the peptides QLCDVMFYL (immunogenic)/QLRDVMFYL (non-immunogenic), EVKEKHEFL (immunogenic)/EVREKHEFL (non-immunogenic), and GLCTLVAML (immunogenic)/GLLTLVAML (non-immunogenic) in the literature (62–66). Compared with non-immunogenic peptides, the third amino acid of the immunogenic peptide is smaller than that in non-immunogenic peptides. The evidence of the peptide pairs appeared to support our hypothesis, and we proposed that the physiochemical properties at P3 could also determine the immunogenicity of a peptide.

**Figure 1:**
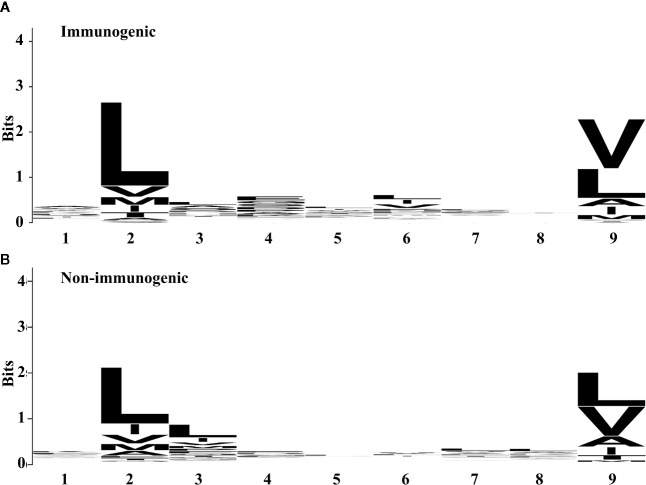
Residue propensity between **(A)** immunogenic and **(B)** non-immunogenic peptides from the training set. The height of amino acid letters within a column indicates the relative frequency of each amino acid at the given position. The overall height of the column indicates the residual conservation at the position. **(A)** and **(B)** were generated by using WebLogo (67).

To investigate the amino acid preferences of the individual position between immunogenic and non-immunogenic peptides further, we compared the frequency of the amino acid at each position (**Figure 1**). Both immunogenic and non-immunogenic peptides had conserved residues, with leucine conserved at P2 and leucine and valine conserved at P9. P3, P4, and P6 had slight differences between immunogenic and non-immunogenic peptides. Based on this finding, the residue propensity value for each amino acid at a specific position was calculated and used as a feature for model construction.

### POTN Construction and Immunogenicity Prediction

**Figure 2:**
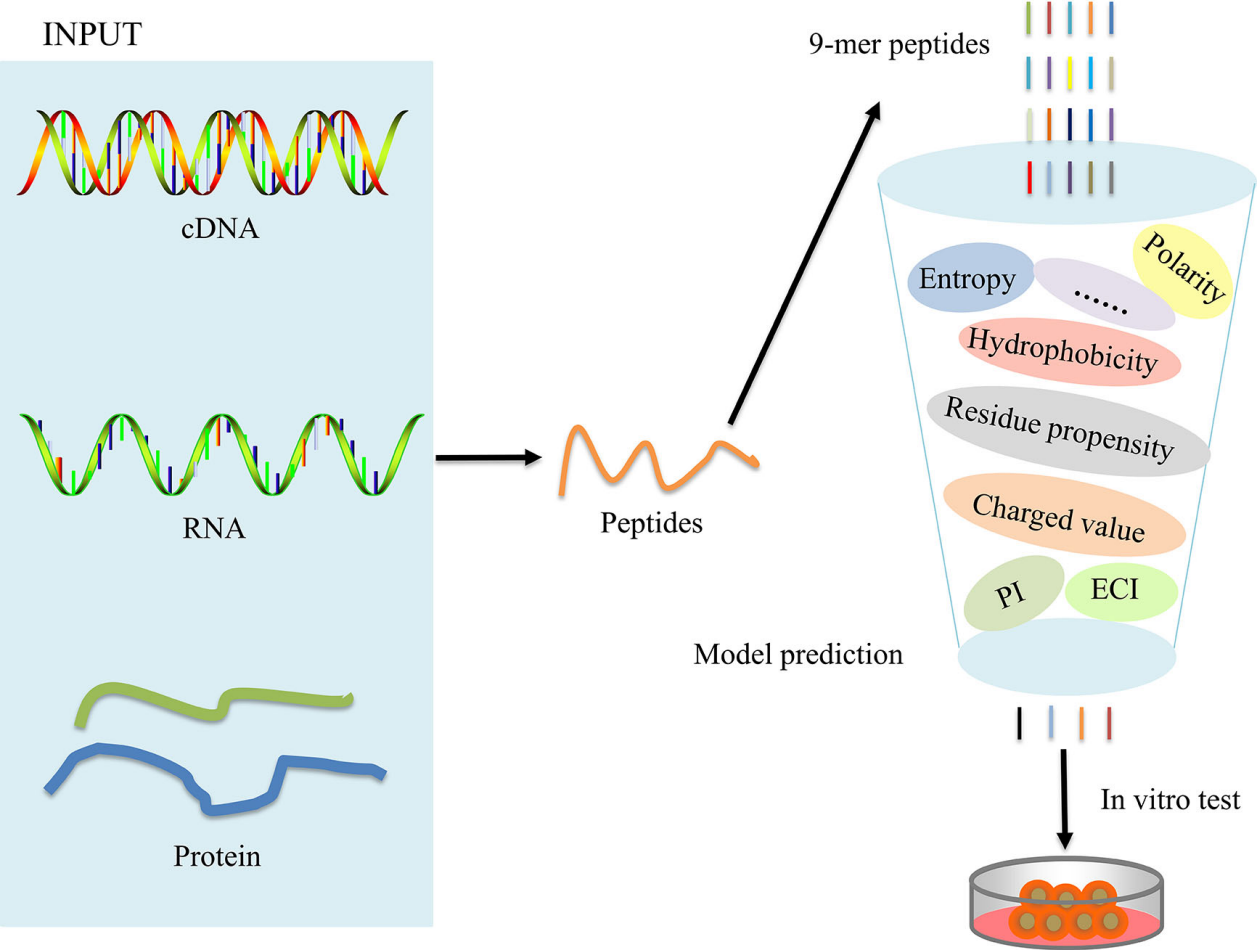
Overall working flowchart for the POTN model construction.

The overall workflow for model construction is shown in **Figure 2**. cDNA, RNA, and amino acids can be processed by POTN, which can split the sequences into nonamers. The model analyzes the properties and calculates the predicted scores, which are used to predict the immunogenicity of peptides. The R implementation of POTN is available in supplementary materials.

**Figure 3:**
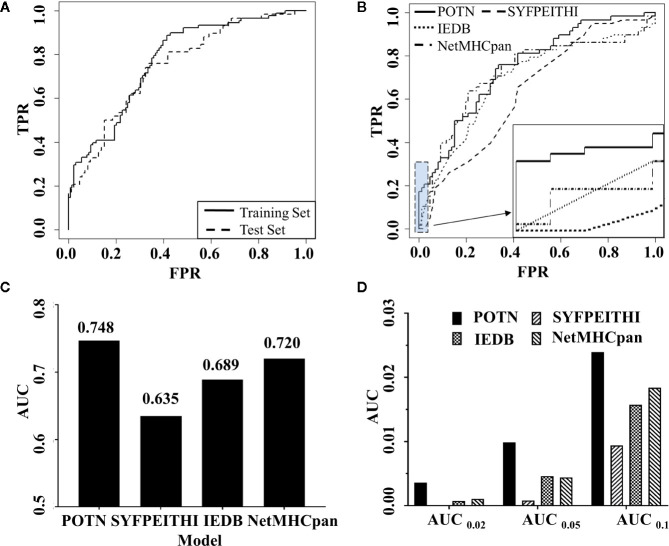
Comparison of the performance of POTN with other programs. **(A)** ROC curves generated by the POTN model with the training set (*n* = 216) and test set (*n* = 144). The black solid line shows the ROC curve for the training set. The short-dashed line shows the ROC curve for the test set. **(B)** ROC curves generated by the POTN (black solid line), SYFPEITHI (short-dashed line), IEDB (dotted line), and NetMHCpan 4.0 (dashed-dotted lines) models with the test set. **(C)** AUC generated by the four models with the test set. **(D)** AUC at different FPR.

In order to construct a high-quality model, the cost parameter C (C value) was continually adjusted until the optimal output was reached by leave-one-out cross-validation experiment, where the C value was set to 1 and the optimal model was called POTN. POTN showed a high prediction power in both the training set and the test set. For the training set, the area under the curve (AUC) was 0.773 and the accuracy (ACC) was 0.653 (**Figure 3A**). For the test set, the AUC was 0.748 and ACC was 0.701.

To illustrate the predictive power of the POTN model further, we compared the predictive power with the prediction programs SYFPEITHI, IEDB, and NetMHCpan 4.0 (**Figure 3B**). The performance of POTN was better than that of the other models with the test set (**Figures 3B, C** **)**. Receiver operating characteristic curves (ROC) based on the four models were plotted. The AUC in the whole test set were 0.748, 0.635, 0.689, and 0.720 for POTN, SYFPEITHI, IEDB, and NetMHCpan 4.0, respectively. The ACC in the whole test set were 0.701 and 0.653 for POTN and NetMHCpan 4.0, respectively. The AUC were also analyzed at different false-positive rates (FPR) (**Figure 3D**). The AUC was 0.01 at an FPR of 0.05 for POTN, which showed the best performance of the prediction models. In addition, we also compared the precision indicator, which was calculated by the ratio of the true positive to the predicted positive peptides. In the test set, the precision indicator of NetMHCpan 4.0 was 54.55%, while the precision indicator of POTN was 67.44%, with 23.63% improvement [the method for improvement rate calculation was referred to (68)].

### Application to CT-X Antigen Dataset

**Figure 4:**
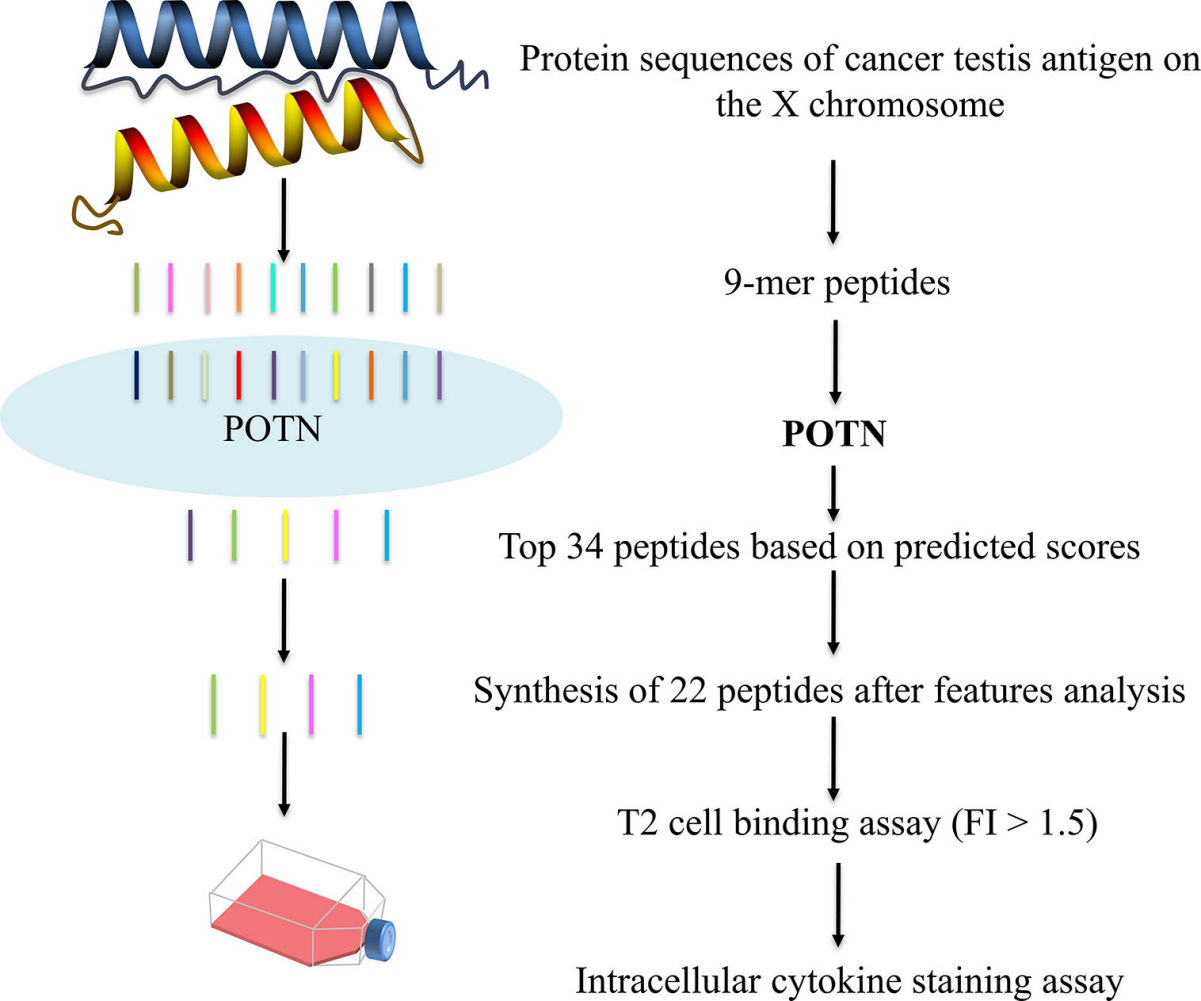
Prediction of immunogenic peptides from CT-X data by using the POTN model and *in vitro* verification.

We applied the POTN model to a dataset of CT-X antigens, which are tumor antigens overexpressed in the testis and other malignancies, as an antigen resource to screen epitope candidates. The amino acid sequences of these antigens were cleaved into nonamers, and POTN obtained a total of 17,310 nonamers from more than 50 antigens after excluding duplicates (**Table S2**) (**Figure 4**). The immunogenic value of each peptide was predicted by POTN, and the top 0.2%, consisting of 34 peptides, was selected based on the predicted values. The solubilities of these 34 peptides were predicted using the MOE package, and 22 of 34 peptides were selected as being sufficiently soluble (**Table 3**).

**Table 3:** Overview of the immunogenicity and HLA-A2 binding affinity of candidate peptides predicted by the POTN model.

Peptide	Prediction score	Binding	Immunogenicity*
KLSSIIPSA	1.1299	++	1/5
FLAKLNNTV	1.1257	+	\
FLSKLSSII	1.1157	**-**	\
VLSAVTPEL	1.1020	+	\
VLSNVLSGL	1.1010	+	\
SIDDLSFYV	1.0988	++	4/5
ILDRANQSV	1.0906	++	3/5
YLATADMPA	1.0898	++	3/5
ALDEKVAEL	1.0847	++	4/5
ALSTVLPGL	1.0832	++	2/5
TLDEKVAEL	1.0777	++	5/5
TLDQVLDEV	1.0680	++	5/5
AMASASPSV	1.0663	++	3/5
VLSTAPPQL	1.0654	++	4/5
KVADLIHFL	1.0653	+	\
KVAELVHFL	1.0627	+	\
LMDVQIPTA	1.0546	++	5/5
ALSVMGVYV	1.0541	+	\
FLAMLKNTV	1.0498	+	\
KVAKLVHFL	1.0493	+	\
KMAGELIKI	1.0386	++	4/5
FIDKLVESV	1.0359	++	5/5

Prediction scores were ranked and retained with four decimal digitals. -, FI < 0.5; +, FI ≥ 0.5 to < 1.5; ++, FI ≥ 1.5; \, no experimental data. *The response ratio of each peptide in five donors detected by the percentages of IFN-γ^+^-secreting CD8^+^ T cells.

**Figure 5:**
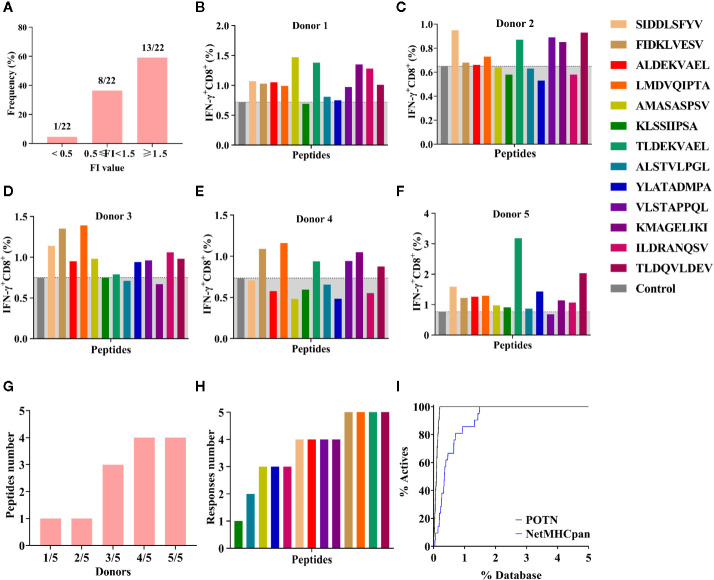
Binding affinity and ICS assay for the peptides identified using the POTN model. **(A)** Identified peptides categorized based on binding affinity to HLA-A2. ICS assay for each peptide in **(B)** donor 1, **(C)** donor 2, **(D)** donor 3, **(E)** donor 4, and **(F)** donor 5, **(G)** Number of immunogenic peptides in each group (e.g., “1/5” indicates that in the group, one peptide elicited T cell response in one donor [1/5]; “2/5” indicates that in the group, one peptide elicited T cell response in two donors [2/5]). **(H)** Number of the donors in which an immune response was elicited by the identified peptides. **(I)** Enrichment curves.

The 22 peptides were synthesized to test the activity. The binding affinity of the synthesized peptides to HLA-A2 was measured *via* a binding assay (FI) with the T2 cell line (27). Based on the FI, the peptides were clustered into three groups: weak binding affinity (FI < 0.5), moderate binding affinity (FI ≥ 0.5 to < 1.5), and high binding affinity (FI ≥ 1.5) (**Figure 5A**). Most of the synthesized peptides (59.09%+36.36%) had a moderate or high FI value (FI ≥ 0.5), of which a large proportion (61.9%) had a high binding affinity and a smaller proportion (38.1%) had moderate binding affinity. Of the 22 synthesized peptides (**Table 3**), eight peptides had moderate binding affinity (FI ≥ 0.5 to < 1.5) and 13 peptides had a high binding affinity (FI ≥ 1.5), which showed that the POTN model had an accuracy rate of 95.45% (21 of 22 synthesized peptides) in predicting the HLA-A2 binding peptides.

Next, we examined the T cell responses of the 13 synthesized peptides with high binding affinity by detecting the percentages of IFN-γ^+^ CD8^+^ T cells from five HLA-A2^+^ healthy donors. A higher percentage of IFN-γ^+^ CD8^+^ T cells in the total CD8^+^ T cell population than that of the negative control indicated an immunogenic peptide. In donor 1, 12 were immunogenic (**Figure 5B**); in donor 2, eight peptides were immunogenic (**Figure 5C**); in donor 3, 10 peptides were immunogenic (**Figure 5D**); in donor 4, six peptides were immunogenic (**Figure 5E**); and in donor 5, 12 peptides were immunogenic (**Figure 5F**). These results showed that more than half of the peptides elicited immune responses in at least three donors, whereas peptide KLSSIIPSA only elicited a response in donor 5 (**Figures 5F, G** **)**. In other words, any of the 13 high-affinity peptides could stimulate a T lymphocyte response in at least one donor (**Figures 5G, H** **)**.

In addition, we compared the virtual screening performance of the POTN model with that of NetMHCpan 4.0. The enrichment curves of the two models showed that both programs efficiently distinguished the immunogenic peptides from the database (**Figure 5I**). All immunogenic peptides were identified in the top 1% of the database by using POTN, and they were identified in the top 2% of the database by using NetMHCpan 4.0. The results indicated that the screening performance of POTN was two-fold better than that of NetMHCpan 4.0.

## Discussion

Cancer immunotherapy has achieved great clinical success, and many studies have shown that the clinical effect depends on the presence of tumor-specific T lymphocytes in patients (69). The tumor-specific T lymphocytes kill tumor cells by secreting cytokines, releasing granzymes, and producing perforin when the MHC-bound peptide is recognized by CTLs. With the development of next-generation sequencing technologies, tumor antigens from cancer patients can be identified easily by sequencing the cancer biopsy. These proteins can be fragmented into numerous peptide sequences, some of which can be presented by the MHC molecule and trigger a specific T cell response targeting the peptide-expressing tumor cells. However, efficiently identifying the MHC binding and immunogenic peptides from the huge amount of sequencing data remains a challenge.

Current programs used for either MHC binding or antigenic prediction are still inaccurate. Possible reasons include the lack of experimental data for many HLA alleles, the non-immunogenic peptides selected for model building include false negatives, and the use of pan-specific methods. To overcome these problems, we designed the POTN model to predict T cell response of peptides to HLA-A2, a common allele of MHC-I.

For current programs, the negative data sets selected for many predictive models are random peptides, which allow some potentially immunogenic peptides to be classified as non-immunogenic. To construct a model with a better predictive effect, 360 nonamers verified by *in vitro* immunological activity experiments were used to construct the POTN model. We selected non-immunogenic peptides with experimental data as our negative data set. These peptides have binding affinity but are not immunogenic, and they have properties that are more similar to the immunogenic peptides. Thus, we chose these peptides as our dataset to identify properties that are directly related to immunogenicity and build a better model.

We collected 216 peptides as the training set and 144 peptides as the test set for the model. To effectively distinguish the MHC binding nonamers from the sequence database, we used all of the peptide features to construct a predictive model. Statistically significant features were selected for model construction. Because the peptides had nine amino acids, these features were further decomposed into 28 descriptors for each peptide (**Table 2**). The relationships between the peptide features and immunogenicity indicated that many features were statistically different at P3, and that P3 may be an important position for distinguishing immunogenicity (**Figure S1**). This result was unsurprising, because the amino acids that came into contact with the MHC/peptide/T cell receptor complex in the nonamers were typically at P4–P7. The features of the amino acid at P3, which is adjacent to sites P4–P7, may indeed be a factor affecting immunogenicity.

The performance of the POTN model was superior to that of the other widely used prediction programs, IEDB, SYFPEITHI, and NetMHCpan 4.0 (**Figure 3B**). The high true positive rate and low false negative rate of the POTN model indicated that it could accurately predict epitopes from a peptide sequence database, which may facilitate the development of personalized cancer immunotherapy based on exome sequencing. The performance of the POTN model proved that the properties of peptides, such as polarity, charges, and entropy, give useful information about how likely it is that a peptide is an epitope, which indicates a new direction for software development.

Antigen presentation is crucial to the function of the adaptive immune response, where the HLA molecule presents the antigenic peptides (epitopes) to T cells and stimulates their proliferation and activation. HLA-A2 is a common allele in humans. Therefore, a prediction model that can specifically identify HLA-A2 epitopes is useful for cancer vaccine development. Our model is designed for this purpose and only predicts epitopes for HLA-A2 (30, 70). Therefore, the current version of the POTN model is restricted to predicting HLA-A2–bound peptides. However, other MHC allele-specific prediction models could be built with the same approach if the experimental binding data for the allele are provided. In addition, only nonamers were evaluated using the model, so the prediction power of the model for peptides with other lengths is not clear. In addition, we wonder about the performance of the POTN system for the peptides from thymic selection. The mechanism of central immune tolerance allows immature T cells of the central immune organ to develop immune tolerance when exposed to self-antigens, and therefore the tolerated self-peptide after thymic selection should not have the characteristics as that from immunogenic peptides, and they can theoretically be excluded by the POTN system. To test the performance of the POTN system for self-peptides, we deliberately selected two self-proteins for study, and the prediction results showed that POTN predicted several self-peptides as immunogenic peptides, although the false positive rates were extremely low (0.36% and 1.7%, separately). The results indicated that the POTN system cannot absolutely exclude self-peptides from immunogenic peptides and the input data for POTN system is suggested to the mutated sequencing data.

Finally, we selected peripheral blood samples from five healthy donors to test the high-affinity HLA-A2 binding peptides, and at least half of the peptides elicited a T cell response in three or more donors. The results showed that anti-tumor immunity could be activated by these peptides in cancer patients, which should be investigated further in an *in vivo* study of tumor treatment with the identified peptides.

## Conclusion

The easy acquisition of personalized exome sequencing data from cancer patients requires a tool for identifying epitopes with high prediction power. In this study, we developed the POTN model to predict the immunogenicity of HLA-A2 peptides, and our model showed superior performance compared with the most commonly used programs, SYPEITHI, IEDB, and NetMHCpan 4.0. POTN may help to identify tumor neoepitopes efficiently from sequencing data, and the approach behind the model may provide a method for constructing prediction models for other MHC alleles. We used the POTN model to identify several epitopes from the CT-X database and four of the peptides elicited a T cell response in all five healthy donors. These peptides could serve as starting points for developing new cancer treatments.

## Data Availability Statement

All datasets generated for this study are included in the article/**Supplementary Material**.

## Ethics Statement

The studies involving human participants were reviewed and approved by: Ethics Committee of Zhengzhou University and Henan Red Cross Blood Center with the approval of the Institutional Ethics Review Board. Written informed consent for participation was not required for this study in accordance with the national legislation and the institutional requirements.

## Author Contributions

YG, YQ, and YW designed the experiments for peptide synthesis, binding assay, and T cell response. JD and QM designed the *in silico* experiments for model construction and data analysis. QM, YW, JM, TW, and YL performed the experiments with critical support from ZW and XZ. XS and QM analyzed the data. QM, JD, and XS wrote the first draft of the manuscript. All authors contributed to the article and approved the submitted version.

## Funding

This work was supported by National Natural Science Foundation of China (Project No. 31500620, U1604286, 81601448), the Henan Province and the Key Scientific Research Projects of Henan Higher Education Institutions (No. 19A180007, 19A180009).

## Conflict of Interest

The authors declare that the research was conducted in the absence of any commercial or financial relationships that could be construed as a potential conflict of interest.
