# Supplementary material for: POTN: A Human Leukocyte Antigen-A2 Immunogenic Peptides Screening Model and Its Applications in Tumor Antigens Prediction
Source: Front Immunol. 2020 Oct 7;11:02193. doi: 10.3389/fimmu.2020.02193 (PMC7579403; doi:10.3389/fimmu.2020.02193)
Supplement: Supplementary file 1 [file DataSheet_1.pdf]

Supplementary Material

Figure Legends

Figure S1. Differences in physicochemical properties between immunogenic (Positive) and non-immunogenic (Negative) peptides.

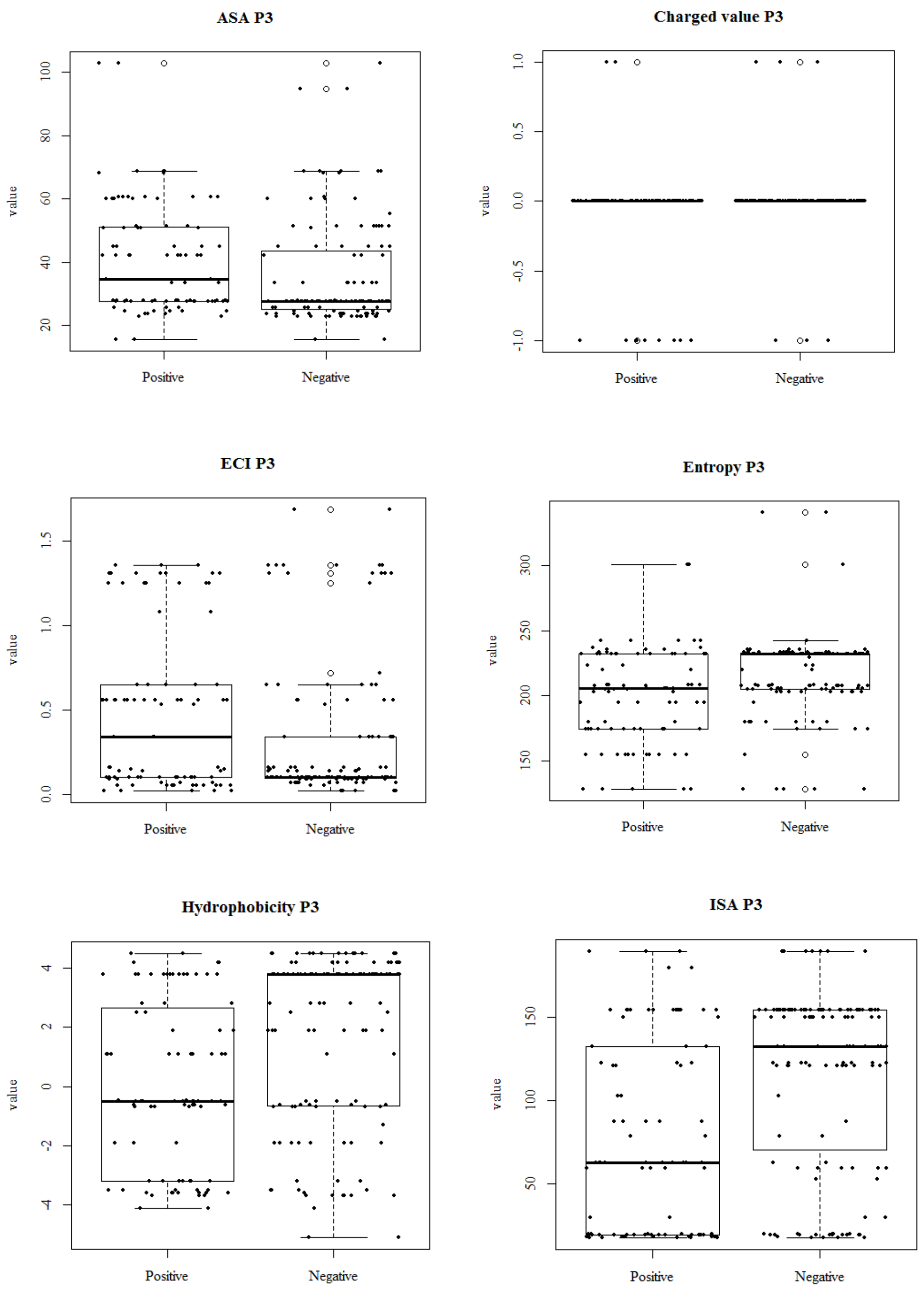

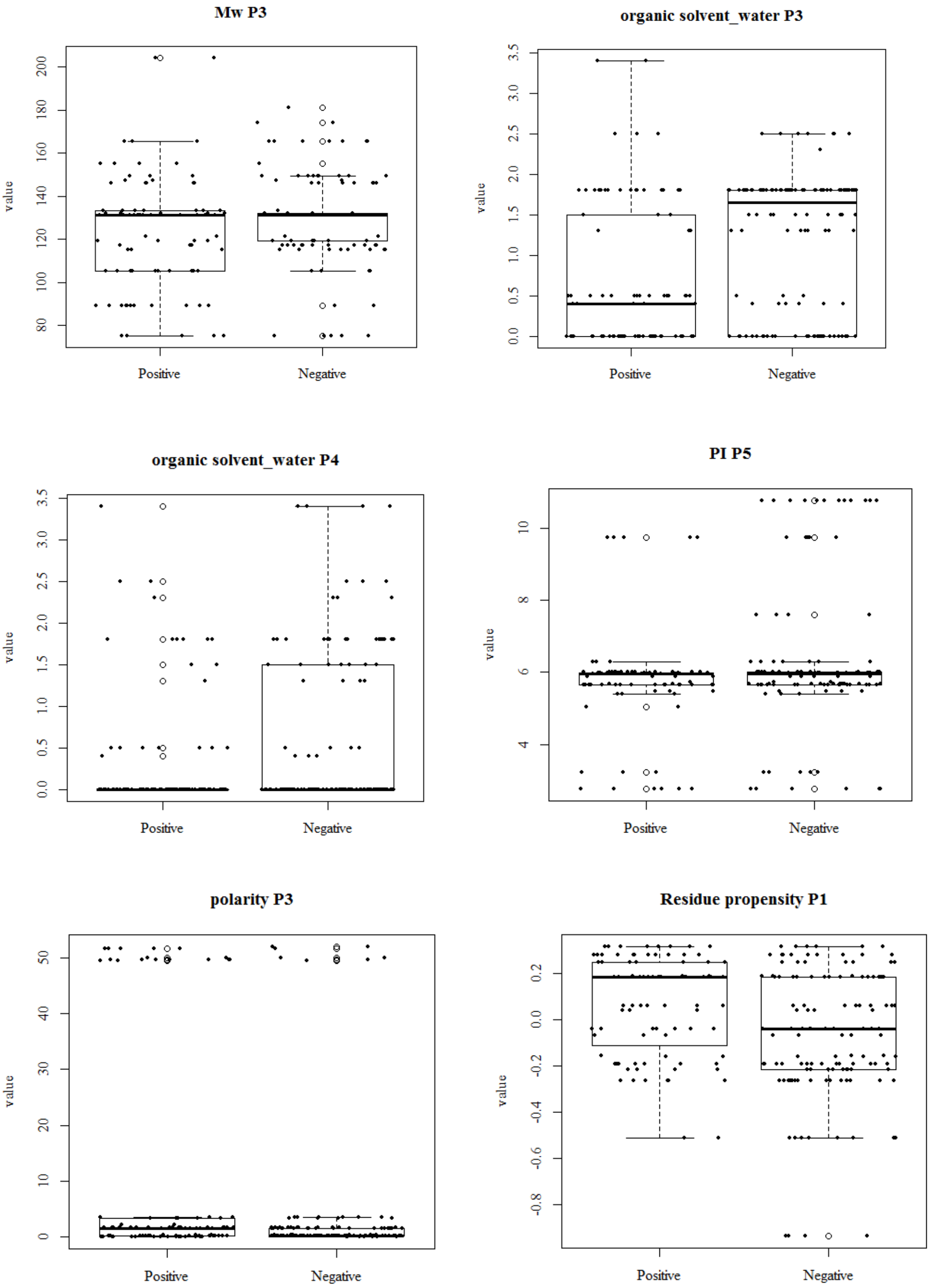
Mw P3
value
80
100
120
140
160
180
200
Positive
Negative
organic solvent_water P3
value
0.0
0.5
1.0
1.5
2.0
2.5
3.0
3.5
Positive
Negative
organic solvent_water P4
value
0.0
0.5
1.0
1.5
2.0
2.5
3.0
3.5
Positive
Negative
PI P5
value
4
6
8
10
Positive
Negative
polarity P3
value
0
10
20
30
40
50
Positive
Negative
Residue propensity P1
value
-0.8
-0.6
-0.4
-0.2
0.0
0.2
Positive
Negative

Residue propensity P2

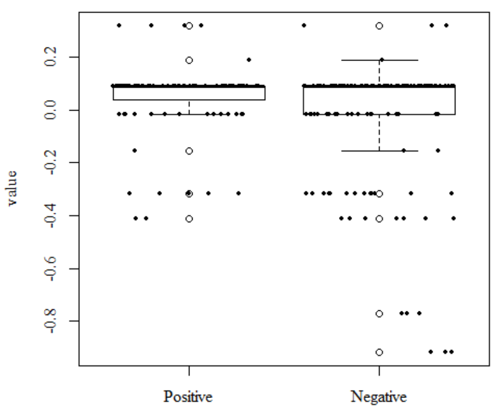

Residue propensity P3

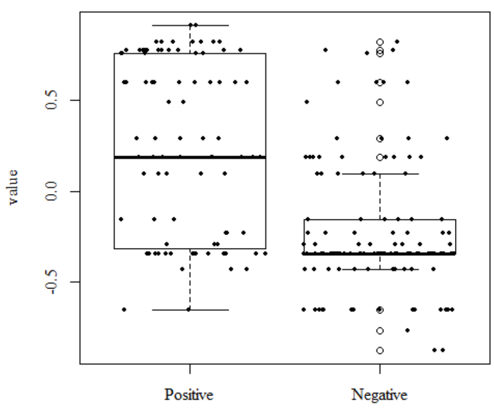

Residue propensity P4

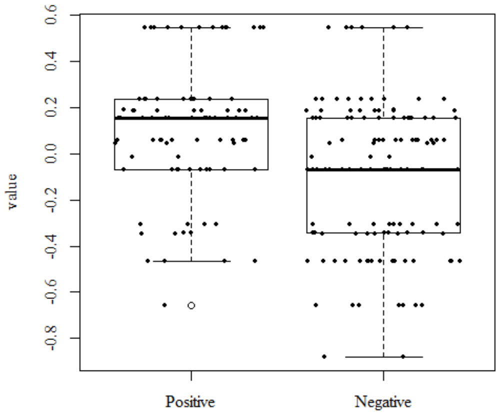

Residue propensity P5

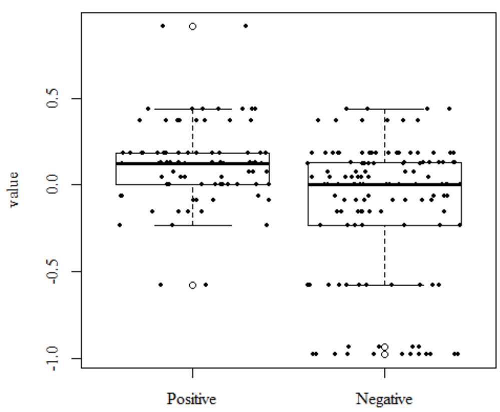

Residue propensity P6

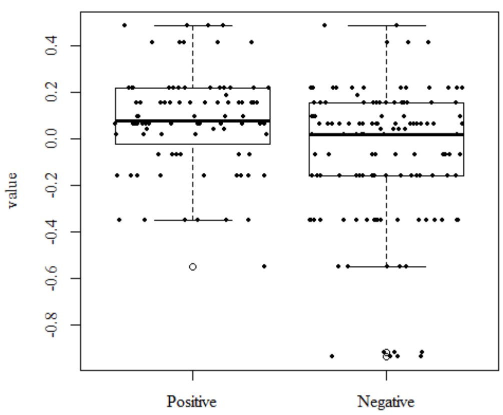

Residue propensity P7

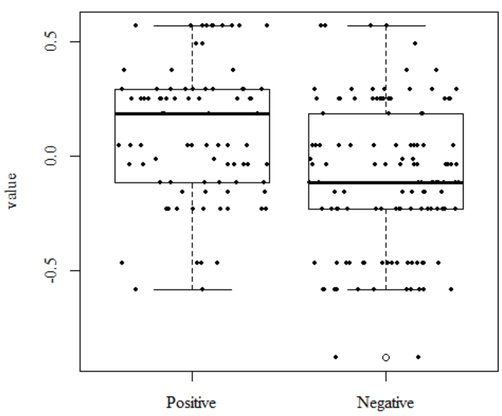

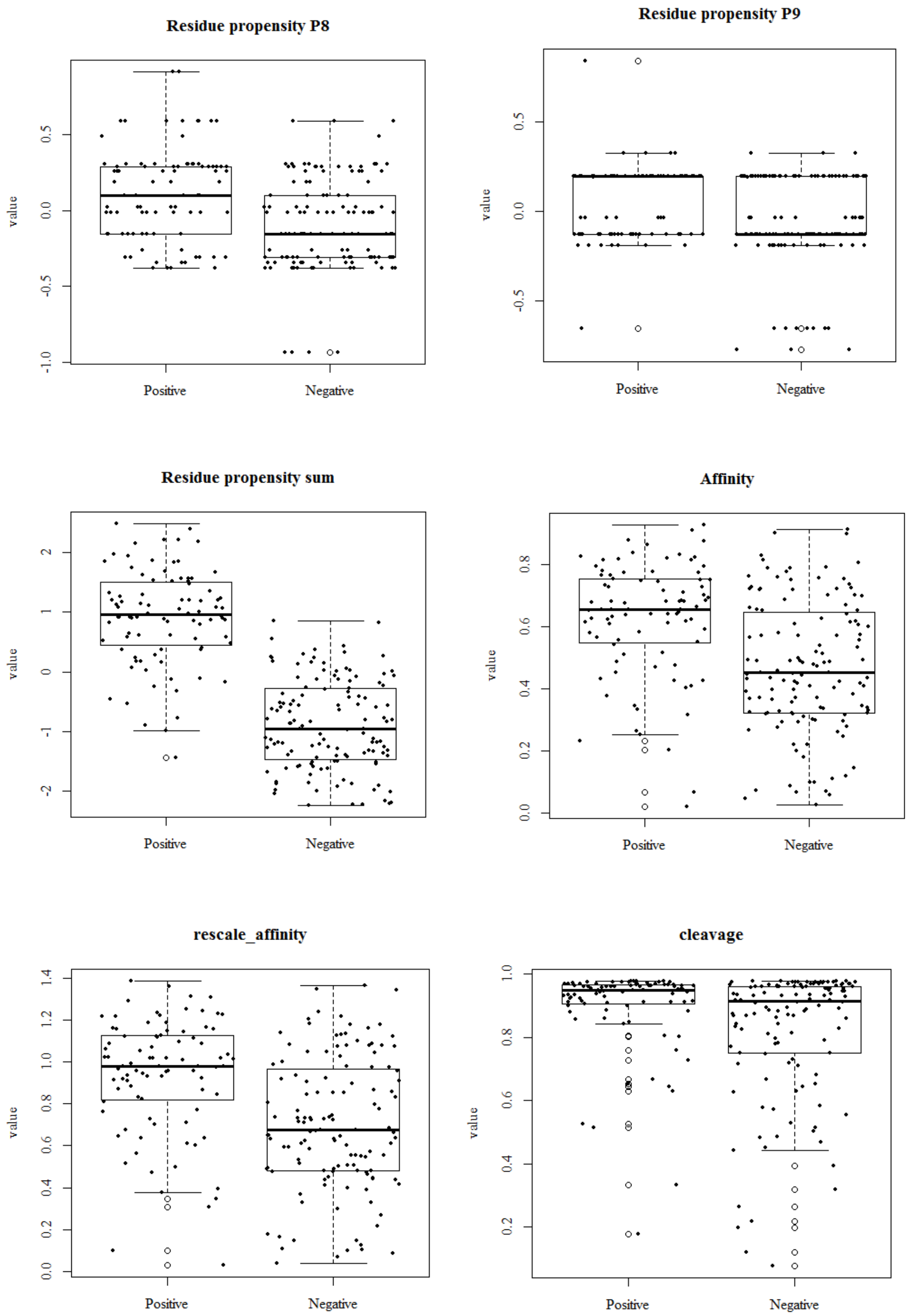
Residue propensity P8
value
-1.0
-0.5
0.0
0.5
Positive
Negative
Residue propensity P9
value
-0.5
0.0
0.5
Positive
Negative
Residue propensity sum
value
-2
-1
0
1
2
Positive
Negative
Affinity
value
0.0
0.2
0.4
0.6
0.8
Positive
Negative
rescale_affinity
value
0.0
0.2
0.4
0.6
0.8
1.0
1.2
1.4
Positive
Negative
cleavage
value
0.2
0.4
0.6
0.8
1.0
Positive
Negative

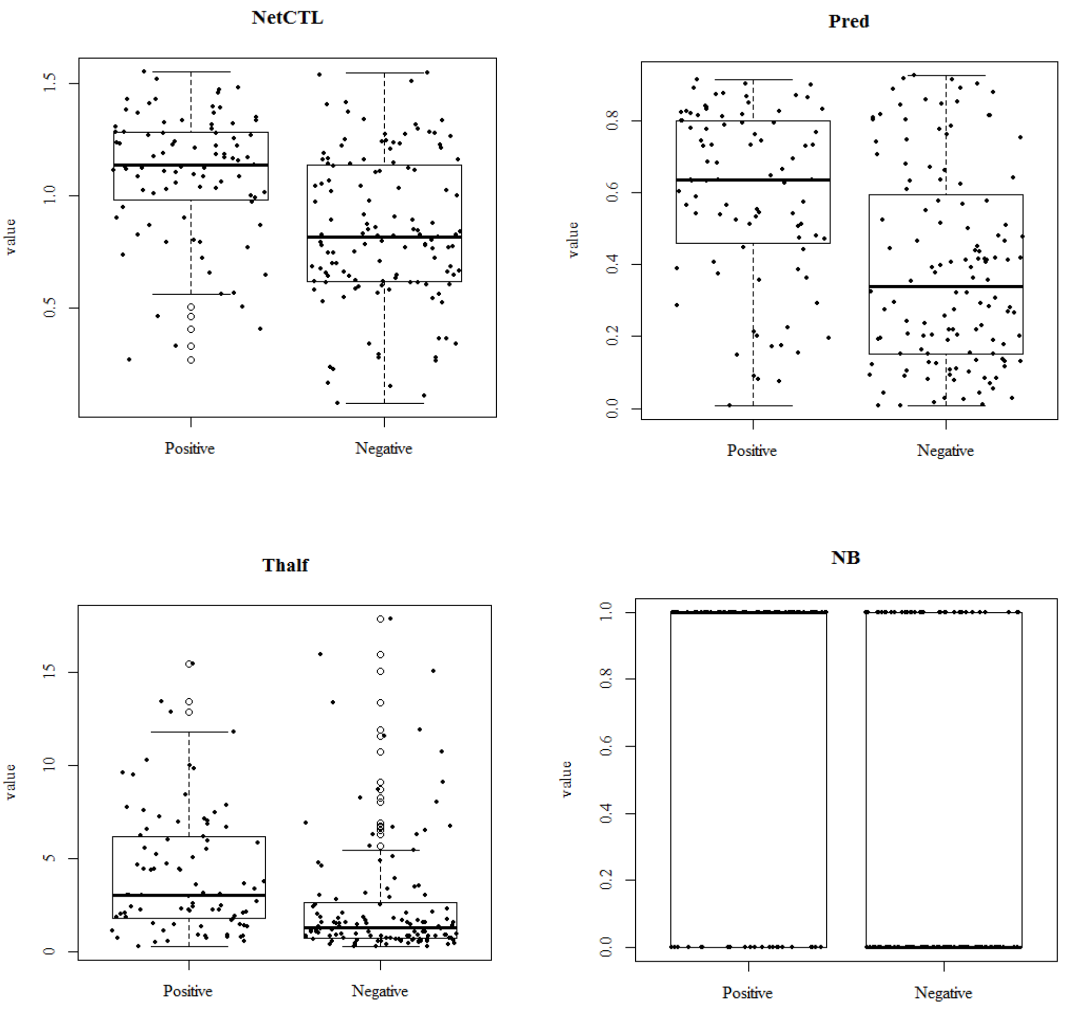
NetCTL
value
1.5
1.0
0.5
Positive
Negative
Pred
value
0.8
0.6
0.4
0.2
0.0
Positive
Negative
Thalf
value
15
10
5
0
Positive
Negative
NB
value
1.0
0.8
0.6
0.4
0.2
0.0
Positive
Negative
